# Was ist das Anthropozän und was wird es gewesen sein? Ein kritischer Überblick über neue Literatur zum kontemporären Erdzeitalter

**DOI:** 10.1007/s00048-020-00269-1

**Published:** 2020-08-12

**Authors:** Andreas Folkers

**Affiliations:** Institut für Soziologie, Justus-Liebig-Universität, Karl-Glöckner-Str. 21E, 35394 Gießen, Deutschland

**Kathryn Yusoff 2019.** ***A Billion Black Anthropocenes or None (Forerunners: Ideas First from the University of Minnesota Press, Band 53).*** Minneapolis, MN: University of Minnesota Press, brosch., 99 S., 1 Abb., 7,95 US$, ISBN: 978-1-5179-0753‑2.

**Paul Warde, Libby Robin und Sverker Sörlin 2018.** ***The Environment: A History of the Idea. ***Baltimore: Johns Hopkins University Press, geb., 256 S., 2 Abb., 29,95 US$, ISBN: 978-1-4214-2679‑2.

**Anna Lowenhaupt Tsing, Heather Anne Swanson, Elaine Gan und Nils Bubandt (Hg.) 2017.** ***Arts of Living on a Damaged Planet: Ghosts and Monsters of the Anthropocene.*** Minneapolis: University of Minnesota Press, brosch., 174 S., 57 Abb., 27,95 US$, ISBN: 978-1-5179-0236‑7.

**Donna J. Haraway 2016.** ***Staying with the Trouble: Making Kin in the Chthulucene.*** Durham: Duke University Press, brosch., 296 S., 31 Abb., 27,95 US$, ISBN: 978-0-8223-6224‑1.

**Christophe Bonneuiland und Jean-Baptiste Fressoz 2016.** ***The Shock of the Anthropocene: The Earth, History and Us.*** London: Verso Books, brosch., 320 S., zahlreiche Abb., 11,99 GBP, ISBN: 978-1-7847-8503‑1.

**Andreas Malm 2016.** ***Fossil Capital: The Rise of Steam Power and the Roots of Global Warming. ***London: Verso Books, geb., 496 S., 70,00 GBP, ISBN: 9781784781323.

## Wissen(schaft) vom und im Anthropozän

„Die Nordwest-Passage verbindet den Atlantik mit dem Pazifik, hoch oben im kalten Norden Kanadas. […] Man umfährt Packeis, Treibeisfelder und Eisberge, schlängelt sich durch kleine Buchten, enge Kanäle, […] und schmale Meerengen.“ (Serres 1994: 15). Michel Serres diente die Nordwest-Passage als Bild, um die Schwierigkeiten zu verdeutlichen, die sich beim Versuch der Überbrückung der „exakten Wissenschaft zur Wissenschaft vom Menschen“ (Serres 1994: 15) ergeben. Heute beginnt das Eis zu schmelzen – sowohl im „Kalten Norden Kanadas“ als auch in den Wissenschaften. Im Zuge des Abschmelzens buchstäblicher und disziplinärer Eisschichten ist mit dem „Anthropozän“ ein neues und bereits dicht besiedeltes Territorium aufgetaucht auf dem sich Kultur‑, Sozial‑, Geistes- und Naturwissenschaften aller Art tummeln.

Bekanntlich geht der Begriff des Anthropozäns auf den Atmosphärenchemiker Paul Crutzen (2002) zurück und bezeichnet ein neues Erdzeitalter, in dem „der Mensch“ zur wichtigsten geologischen Kraft geworden ist. Crutzens Vorschlag hat zu einer ernsthaften Debatte über Sinn und Unsinn des Anthropozäns als geologische Kategorie geführt (Zalasiewicz et al. 2017). Aber auch in den Sozial- und Kulturwissenschaften hat die Debatte um das Anthropozän verfangen und vielfältige Beiträge unter anderem in der Soziologie (Henkel & Laux 2018; Latour 2017; Schroer 2015), Geschichtswissenschaft (Bonneuil & Fressoz 2016; Chakrabarty 2009), Politikwissenschaft (Rothe 2017), Rechtswissenschaft (Kersten 2014; Pottage 2019), Medienwissenschaft (Parikka 2014; Wark 2015), Humangeographie (Wakefield 2014; Clark & Yusoff 2017), Literaturwissenschaft (Horn 2016), Kulturanthropologie (Haraway et al. 2016; Tsing 2015) und Kunstwissenschaft (Davis & Turpin 2015) provoziert.

Diese Begeisterung für das Anthropozän in den Sozial- und Kulturwissenschaften ist kaum verwunderlich. Schließlich geht es ja nicht nur um die Erde, sondern eben auch um den Menschen, seine Gesellschaft und Kultur. Und selbst in die Naturwissenschaften schleicht sich neuerdings eine spontane Anthropologie ein. Was dabei als „Anthropos“ des Anthropozäns präsentiert wird, hat bisweilen erstaunliche Ähnlichkeiten mit der Figur des Menschen, die Michel Foucault (1974) in *Die Ordnung der Dinge* als Subjekt/Objekt der modernen Humanwissenschaften identifiziert hat: ein historisches Wesen, das sich selbst in den Produkten seiner Arbeit, seiner Sprache, seines Lebens erkennt. Der Unterschied ist jedoch, dass genau das, was der „epistemologische Habitus des 19. Jahrhunderts“ (Sloterdijk 2015: 29) noch als Kraft des Menschen und seiner Emanzipation von der Natur gefeiert hatte, nun als große Krise der Menschheit wahrgenommen wird.

Allerdings finden sich in der *episteme* des Anthropozäns auch Elemente, die sich signifikant vom klassischen epistemologischen Niveau der Humanwissenschaften unterscheiden. Das wird vor allem mit Blick auf die Datierungsproblematik klar mit der sich die Geologie aktuell herumschlagen muss. Denn, egal, wann man den Beginn des Anthropozäns ansetzt: Das neue Erdzeitalter ist noch gar nicht alt genug, um bereits einen entsprechend eindeutigen „geological record“ gebildet zu haben (Pottage 2017). Es wird daher immer wieder auf die Denkfigur einer „future geologist“ (Yusoff 2016: 4) zurückgegriffen, die erst in der Zukunft entscheiden kann, ob wir gegenwärtig im Anthropozän gelebt haben werden oder eben nicht. Nun ist es aber durchaus möglich, dass zu dem Zeitpunkt, an dem ausreichende geologische Ablagerungen des Menschlichen vorliegen, die biologische Existenz der Menschheit bereits Geschichte sein wird. Entsprechend werden „future geologists“ nicht selten als Aliens oder künstliche Intelligenzen vorgestellt, die nur noch aus den Überresten des Menschlichen auf dessen planetarische Wirksamkeit schließen können. „Der Mensch“ wäre dann also nicht mehr Subjekt, sondern nur noch Objekt des Wissens. Es muss eine nicht-menschliche Beobachtungsinstanz ins Spiel gebracht werden, die das Menschliche mit objektiver Distanz erkennen kann. Wir haben es also nicht mehr mit einer „Analytik der Endlichkeit“ (Foucault 1974: 377–383) zu tun, sondern mit einer Epistemologie *Nach der Endlichkeit* (Meillassoux 2008), in der das Humane aus den „technofossilen“ Überresten des Menschen rekonstruiert werden muss. Dabei sind diese Technofossilien, die „far-future signals of the Anthropocene“ (Zalasiewicz et al. 2014: 37) nicht so sehr die stolzen Produkte menschlicher Arbeit, sondern eher die Abfälle, die bei dieser Arbeit entstehen: CO2, Plastikabfall (Westermann 2020), Atommüll (Hecht 2018), Hühnerknochen (Patel & Moore 2017: 1) und dergleichen. Nicht *work*, sondern *waste* wird damit zur Signatur des Menschlichen, gewissermaßen zu einer Plastikflaschenpost, die schon gar nicht mehr an menschliche Leser*innen adressiert ist und bei der, so viel steht fest, das Medium die Botschaft ist. Menschen mögen zwar neuerdings als Autor*innen des „Buchs der Natur“ gelten. Zugleich verlieren sie aber ihre Rolle als souveräne Interpret*innen der Erdgeschichte und ihrer selbst. „Der Mensch“ verschwindet nicht einfach wie ein Gesicht im Sand, sondern bleibt gespenstisch anwesend als an den Strand gespültes Treibgut. In diesem Sinne ist die Frage nicht nur: was ist das Anthropozän, sondern: was wird es gewesen sein?

Derartige Spekulationen mögen allzu sehr nach *science fiction* klingen. Gleichwohl deuten sie auf bereits wirksame Rekonfigurationen kontemporärer epistemischer Regime hin. Tatsächlich ist die Wissenschaft vom und im Anthropozän von mehr-als-menschlichen Beobachtungsagenzien bevölkert, die menschliches Handeln detektieren und sichtbar machen: Satelliten, die den Planeten vermessen (Jasanoff 2004), Sensoren, die noch an den entlegensten Orten Daten aufzeichnen (Gabrys 2016), Computer, die diese Flut von Daten aufbereiten und zu einem digitalen Weltbild zusammenführen (Edwards 2010). Diese „Wissensinfrastrukturen des Anthropozäns“ (Edwards 2015) sind nicht nur für die großen Durchbrüche in Klima- und Erdsystemwissenschaft verantwortlich. Sie bestimmen schon heute immer stärker die Politik, das Wirtschaften und das Alltagsleben. So banale Devices wie intelligente Stromzähler, die über den CO2-Fußabdruck des verbrauchten Stroms informieren, sind Teil einer neuen epistemologischen Realität, in der alltägliches und lokales Handeln von digitalen Technologien auf seine planetarischen Konsequenzen hin abgeklopft wird und diese dann an die Handelnden zurückgespielt werden (Folkers & Marquardt 2018).

## Ursprünge und Urheberschaften des Erdzeitalters

Trotz dieser Hybridisierung der Wissensformen lösen sich keineswegs sämtliche disziplinären Unterschiede auf. Das zeigt sich insbesondere in der Debatte um die richtige Datierung des neuen Erdzeitalters, die eng mit der Frage verknüpft ist, ob das „Anthropozän“ nicht vielleicht doch mit einem anderen Namen belegt werden sollte. Denn Ursprungsfragen hängen hier eng mit der Frage nach Urheberschaft zusammen. In den Naturwissenschaften werden gegenwärtig vor allem drei Datierungsvorschläge intensiv diskutiert (Zalasiewicz 2015). Crutzens (2002) Vorschlag lässt das Anthropozän mit der Erfindung der kohlebetriebenen Dampfmaschine Ende des 18. Jahrhunderts beginnen. In dieser Fokussierung auf den Konnex von Dampfkraft und fossilen Brennstoffen wird der Klimawandel als zentrale Krise des Anthropozäns inszeniert. Denn durch den Eingriff menschlicher Technik ist eine komplette geologische Mineralschicht in die Atmosphäre geblasen worden. Die „geologisch“ relevante Inskription des Menschlichen findet sich also nicht nur in Erd- und Gesteins-, sondern auch in Atmosphärenschichten. Die Popularität dieses Datierungsvorschlags zeigt, dass längst nicht nur Geolog*innen *stakeholder* in der Anthropozändiskussion sind. Insbesondere die Erdsystemwissenschaften (Schellnhuber 1999), ein interdisziplinäres Forschungsfeld, das Erkenntnisse aus Biologie, Physik und Chemie miteinander in Bezug bringt, ist hier von besonderer Bedeutung.

Einem alternativen Vorschlag zufolge beginnt das Anthropozän wesentlich früher, nämlich mit dem Aufkommen der Menschheit (Zalasiewicz 2015: 166). Schon die gezielte Nutzung von Feuer (Clark 2012), die Jagd auf Großtiere und die Bearbeitung der Erde seit der Entwicklung des Ackerbaus habe die Umwelt so umfassend transformiert, dass es gerechtfertigt sei, bereits hier das Anthropozän beginnen zu lassen. Demgegenüber verlegt ein dritter Vorschlag den Beginn des Anthropozäns in die Nachkriegszeit und die zu dieser Zeit einsetzende „great acceleration“, in der die Weltbevölkerung und das Wirtschaftswachstum, aber eben auch die CO2-Emissionen, der Ressourcenverbrauch und das Artensterben exponentiell angestiegen sind (Steffen et al. 2015). Das erdölgetriebene Automobil und die durch das Haber-Bosch-Verfahren ermöglichte Verbreitung von Kunstdünger werden in diesem Zusammenhang als Triebkräfte der großen Beschleunigung identifiziert. Damit gelten wiederum vor allem technische Neuerungen als ausschlaggebendes Moment des Anthropozäns.^1^ Ausgeblendet bleiben damit die komplexen sozialen Umwälzungen in der Zeit nach dem Zweiten Weltkrieg, wie die Dekolonisierung der Länder des Globalen Südens, die Fortschritte in der Emanzipation von Frauen, die Entwicklung des fordistischen Klassenkompromiss in Ländern des globalen Nordens sowie die Systemkonkurrenz zwischen Kapitalismus und Staatssozialismus.

Die Erdsystemwissenschaftler Simon Lewis und Mark Maslin (2015) haben einen weiteren Datierungsvorschlag unterbreitet, der in den naturwissenschaftlichen Debatten allerdings weitaus umstrittener ist (Hamilton 2015) und weniger Resonanz ausgelöst hat. Sie argumentieren, dass das Anthropozän um 1600 mit dem sogenannten „Columbian exchange“ (gemeint ist der globale „Austausch“ einer Vielzahl von Tier- und Pflanzenarten) beginnt. Für sie ist also der moderne Kolonialismus die entscheidende Triebkraft für den Beginn des Anthropozäns, weil „die Menschheit“ nun im planetarischen Maßstab zur entscheidenden Wirkkraft im Gewebe der Natur wird. In ihrem Bemühen um vermeintliche naturwissenschaftliche Neutralität neigen Lewis und Maslin dabei zwar gewiss zu einer Verharmlosung kolonialer Machtverhältnisse als „Austausch“ und von Prozessen imperialistischer Landnahme als „Aufeinandertreffen“ (Yusoff 2018: 29–33). Gleichwohl verschweigen sie die Gewalt des Kolonialismus nicht, sondern zeigen, wie sich diese in die Erdgeschichte eingeschrieben hat. Der koloniale Genozid in Amerika hat zu einer Reduzierung des CO2-Flusses in die Atmosphäre beigetragen und lässt sich so noch heute in Eisbohrkernen nachweisen (Lewis & Maslin 2015). Erdgeschichtliches Wissen wird so zum Zeugnis der Grausamkeiten der Menschheitsgeschichte.

Der Vorschlag von Lewis und Maslin nimmt so eine Brückenfunktion zu Beiträgen aus den Sozial- und Kulturwissenschaften wahr, die ebenfalls die Rolle des Kolonialismus für die Entwicklungen eines neuen Erdzeitalters betonen. Allerdings wird in vielen dieser Beiträge der generische Bezug auf „den Menschen“ kritisiert, weil dadurch soziale Asymmetrien ebenso wie sozio-ökonomische Triebkräfte der Geschichte ausgeblendet werden. Entsprechend werden alternative Namen für das neue Erdzeitalter erprobt, die besser in der Lage sein sollen, die Wirkmechanismen und Akteur*innen der großen sozial-ökologischen Transformation zu benennen. Das Konzept des „Plantationocene“ geht zurück auf eine Diskussion an der unter anderem Anna Tsing und Donna Haraway teilgenommen haben, (Haraway et al. 2016). Das Plantationozän ist ein Gegenentwurf zum Westeuropa-zentrierten Anthropozänvorschlag von Crutzen und betont, dass die Kombination aus Sklav*innenarbeit und landwirtschaftlicher Monokultur auf den kolonialen Plantagen eine entscheidende Voraussetzung und Blaupause für die fossile Industrialisierung Westeuropas war. „I think we are looking at slave agriculture, not coal, frankly, as a key transition“ (Haraway et al. 2016: 555; siehe auch: Haraway 2016: 99–103). Wie Anna Tsing (2015: 40) am Beispiel der kolonialen Zuckerrohrplantagen erläutert, hat das Zusammenkommen von „cloned planting stock, coerced labor, conquered and thus open land“ eine sozial wie ökologisch folgenschwere kapitalistische Wirtschaftsweise erzeugt, die darauf basiert, Menschen und Nicht-Menschen aus bestehenden Relationszusammenhängen herauszulösen und zu abstrakten Kapitalgütern zu degradieren. Zwar lassen sich Tsing und Haraway zurecht dafür kritisieren, dass sie zu wenig auf bestehende Arbeiten zu Plantagenarbeit und Sklaverei hingewiesen haben (siehe etwa: McKittrick 2013) und dazu tendieren, die Unterschiede zwischen der Ausbeutung und Entfremdung von Menschen- und Nicht-Menschen einzuebnen (Davis et al. 2019). Der Versuch, die Naturgeschichte des Anthropozäns enger mit der Sozialgeschichte der Moderne zu verknüpfen, ist jedoch ebenso bedeutsam, wie der Hinweis auf all die Menschen, die den Preis für die Entwicklung des Anthropozäns schon lange vor den weithin anerkannten ökologischen Katastrophen der Gegenwart zahlen mussten: von den Arbeiter*innen in den Kohleminen Westeuropas bis zu den Sklavenarbeiter*innen auf den Zuckerrohrplantagen der Karibik.

In ihrem *A Billion Black Anthropocenes or None* geht Kathryn Yusoff (2018) noch einen Schritt weiter, indem sie die in den hegemonialen Anthropozänerzählungen so selbstverständlich verwendete Kategorie des „Menschen“ mithilfe der *black studies *kritisiert, die hegemoniale Konzeptionen des Humanen radikal in Frage gestellt haben (Wynter 2003). Yusoff argumentiert, dass das moderne Verständnis des Menschen nur durch den dehumanisierenden Akt des Ausschlusses von *people of colour* aus der Gemeinschaft des Menschlichen gebildet werden konnte. Die moderne Unterscheidung Mensch vs. Nicht-Mensch hat damit nicht nur die Ausbeutung der Natur, sondern auch die Versklavung, „Extraktion“ und „Transplantation“ afrikanischer Menschen ermöglicht. Entsprechend datiert sie den Beginn des Anthropozäns der jamaikanischen Theoretikerin Sylvia Wynter folgend auf das Jahr 1452, als afrikanische Sklav*innen auf den ersten Zuckerrohrplantagen der Portugiesischen Insel Madeira zur Arbeit gezwungen wurden (Yusoff 2018: 33–39).

Auch das „Kapitalozän“-Konzept des öko-marxistischen Theoretikers Jason Moore (2015) bringt das Aufkommen eines neuen Erdzeitalters mit machtgeladenen Zäsuren sowohl zwischen Natur und Kultur, als auch innerhalb des Menschlichen in Verbindung. So argumentiert Moore, dass der Kapitalismus ein ontologisches Regime sei, das zwar einerseits so tief mit dem *web-of-life* verzahnt ist, dass eine Unterscheidung von Natur und Kultur aus theoretischer Perspektive keinen Sinn mache. Andererseits brauche der Kapitalismus aber genau diese Unterscheidung, um seine Reproduktion zu sichern. Die Akkumulation des Kapitals verdankt sich nämlich – so Moore – nicht nur der Ausbeutung (*exploitation*) von Lohnarbeit, sondern vor allem auch der Aneignung (*appropriation*) von unbezahlter Arbeit, was für ihn sowohl die Reproduktionsarbeit von Frauen, die Arbeit von Sklav*innen als auch die „Arbeit“ der Natur beinhaltet. Das Kapitalozän hat damit seinen Ursprung im langen 16. Jahrhundert, in dem sich das kapitalistische Weltsystem und gleichzeitig die moderne Unterscheidung von Natur und Kultur entwickelte. Aus der Perspektive des Kapitalozäns ist die Dampfmaschine daher nicht die treibende Kraft, sondern lediglich ein Effekt der Entwicklung des Kapitalismus. Das hat Andreas Malm (2016), der als erster den Begriff des Kapitalozäns verwendete, in seiner Studie *Fossil Capital* eindrucksvoll gezeigt. Die kohlegetriebene Dampfkraft konnte sich, so Malm, in der Industrialisierung Großbritanniens gegen die Konkurrenz der Wasserkraft nämlich vor allem aufgrund ihrer besseren Passung zur Logik des Kapitals durchsetzen. Rassismus- und Kapitalismuskritische können also ebenso wie feministische Perspektiven (Merchant 1987) zeigen, dass die Unterscheidung von Natur und Kultur den scheinbar homogenen Bereich des Menschlichen selbst durchzieht, so dass in der kapitalistischen Moderne die Ressourcen der Natur ebenso zur Ausbeutung freigegeben werden konnten, wie die Körper von nicht-Weißen, Frauen und Proletarisierten.

Im Gegensatz zu allen bisher besprochenen Erdgeschichten ist Donna Haraways „Chthulucene“, das sie in ihrem *Staying with the Trouble* eingeführt hat, eher ein Antikonzept, bei dem es darum geht, bestehende Konzeptionen zu irritieren und Alternativen zu den sozial-ökologischen Verheerungen der Gegenwart zu artikulieren. Das Chthulucene „resists figuration and dating and demands myriad names“ (Haraway 2016: 51). Es geht ihr dabei um eine radikale Dezentrierung „des Menschen“ als entscheidendes Subjekt der Erdgeschichte. „Unlike the dominant dramas of Anthropocene and Capitalocene discourse, human beings are not the only important actors in the Chthulucene“ (Haraway 2016: 55). Dafür macht sich Haraway das symbiotische Weltbild zu eigen, das sich gegenwärtig in der Biologie herausbildet (Margulis 1999; Folkers & Opitz 2019; McFall-Ngai 2017; Gilbert 2017), demzufolge nicht eine Spezies die Erde im Griff haben kann, sondern nur „sympoietische“ Bündnisse miteinander zusammenhängender (Lebe)wesen (Haraway 2016: 58–98). In der symbiogenetischen Erdgeschichte haben Menschen weder das erste und gewiss nicht das letzte Wort. Vielmehr steht das Chthulucene für eine kaum in abgrenzbare Zeiteinheiten einteilbare „ongoing temporality“ (Haraway 2016: 51) bzw. eine „ongoing terran finitude“ (Haraway 2016: 53) in der Vergangenheit, Gegenwart und Zukunft ineinander eingehen. Um die komplexen *Zeitschichten* (Koselleck 2000) des Chthulucene anschaulich zu machen, ruft Haraway die Figur des Komposthaufens auf, bei dem Neues aus der Vergärung des Alten entsteht. Insofern weist das Chthulucene über das Anthropozän nicht deshalb hinaus, weil es als Platzhalter für eine utopische Zukunft fungiert, die sich ein für alle Mal von der schmerzhaften Herrschafts- und Ausbeutungsgeschichte des Anthropozäns losgesagt hat, sondern weil das Chthulucene als Komposthaufen der Erdgeschichte diese schmerzhaften Ablagerungen gewissermaßen zu verdauen hat.

## Die politische Epistemologie des Anthropozäns

Peter Sloterdijk (2015: 26) hat den politischen Einsatz der Anthropozändebatte deutlich gemacht, indem er betont hat, dass diese weniger eine rein „geowissenschaftliche“ Diskussion sei, sondern vielmehr eine „Gerichtsverhandlung […] – genauer […] eine Vorverhandlung […] bei welcher fürs Erste die Schuldfähigkeit des Angeklagten geklärt werden soll“. Die Entscheidung darüber, wie das neue Erdzeitalter datiert und benannt wird, hängt damit zusammen, wer oder was für die ökologischen Probleme der Gegenwart verantwortlich ist und damit implizit auch, wie eine angemessene Reaktion darauf aussehen kann. Epistemologische Fragestellungen werden dadurch ethisch aufgeladen, geologische und erdsystemwissenschaftliche Erkenntnisse werden gewissermaßen zur Forensik in der politischen Frage, wer die Verantwortung für die ökologische Krise der Gegenwart trägt (Pottage 2017). In der hegemonialen Erzählung, die Christophe Bonneuil und Jean-Baptiste Fressoz (2016: 45–96) in ihrem überaus lesenswerten *The Shock of the Anthropocene* treffend als „geocratic grand narrative of the Anthropocene“ bezeichnen, wird „die Menschheit“ zum Subjekt der Erdgeschichte erklärt und damit auch für das Wohl der Zukunft des Planeten in die Verantwortung genommen. Wie Bonneuil und Fressoz zeigen, liegt der entscheidende strategische Einsatz der geokratischen Erzählung jedoch darin, die menschliche Schuldfähigkeit zu relativieren. Denn erst die von der Wissenschaft über ökologische und geologische Veränderungen aufgeklärte Menschheit könne als vollumfänglich verantwortlich gelten. So können sich die Erdsystemwissenschaftler*innen als entscheidende Akteur*innen der Erdgeschichte inszenieren, die es ermöglichen, dass „die Menschheit“ nicht nur „an sich“, sondern auch „für sich“ zum Subjekt des Anthropozäns wird.

Interessanterweise stößt auch Bruno Latour, wenn auch in gewohnter Virtuosität, ins gleiche Horn wie die geokratische Erdsystemwissenschaft. Latour (2017: 29) schließt an Haraways (2016: 16) Konzept der „response-ability“ an, die damit die Voraussetzungen betont, die erfüllt sein müssen, um in die Lage versetzt zu werden („rendering capable“– (Haraway 2016: 16)) antworten und nicht bloß reagieren zu können.^2^ Latour gibt diesen Überlegungen allerdings eine kybernetische Wendung. So argumentiert er, dass erst das technisch generierte Feedback über die ökologischen Auswirkungen menschlichen Handelns diese Ver-Antwortungsfähigkeit erzeuge. Die „Wissenschaftsinfrastrukturen des Anthropozäns“ (Edwards 2015) werden damit zugleich zur Infrastruktur ökologischer Moral bzw. politischer Ökologie. In einem jüngeren Artikel, den Latour gemeinsam mit dem Erdsystemwissenschaftler Timothy Lenton in *Science* veröffentlicht hat, spinnt er diese Idee weiter. Die informatorisch mit der Erde verdrahtete Menschheit wird hier als erdhistorischer Evolutionsschritt präsentiert, der zur Emergenz einer „gaia 2.0“ geführt habe – also einem Erdsystem, das sich nicht nur selbst regulieren (Lovelock & Margulis 1974), sondern dies im Sinne einer „self-aware self-regulation“ auch bewusst tun kann (Lenton & Latour 2018: 1067).

So wird ein aus der Unmündigkeit befreites, universelles Menschensubjekt beschworen, das gemeinsam seine Verantwortung für die Erde wahrnehmen muss. Diese Denkfigur gipfelt dann nicht selten in Forderungen nach einer „Planetary Stewardship“ (Steffen et al. 2011) bzw. einer „Earth System Governance“ (Biermann et al. 2012), also in Konzepten, die als gouvernementales Skript verstanden werden können und die neuen Formen der „geopower“ (Bonneuil & Fressoz 2016; Luisetti 2018) Vorschub leisten. Dabei handelt es sich in gewisser Weise um eine Fortsetzung und Ausweitung der Umweltpolitik, die sich im Verlauf des 20. Jahrhunderts entwickelt hat (Warde et al. 2018). Das Anthropozän steht in der Geschichte des Konzepts der „Umwelt“ (Sprenger 2019) und in den Versuchen seiner Regulierung, wie Warde, Robin und Libby (2018) in ihrem *Environment. A History of the Idea* zeigen, dabei vor allem für eine räumliche Ausweitung. „Die Umwelt“ umfasst jetzt nämlich die gesamte Ökosphäre und nicht mehr nur den „Himmel über der Ruhr“, diesen oder jenen Fluss, den lokalen Boden etc. Vielmehr geht es um die vielfältig miteinander verflochtenen ökologischen Relationen im Erdsystem (Lenton et al. 2008), die gemeinsam als „life-support system“ (Young & Steffen 2009) der Menschheit dienen. Ähnlich wie Bonneuil und Fressoz liefert auch das Buch von Warde, Robin und Libby einen Rundumschlag über entscheidende Etappen der jüngeren Geschichte der „Umwelt“. Was sich daraus lernen lässt ist, dass das Wissen über „die Umwelt“ – diesen recht jungen Gegenstand von Wissenschaft und Politik – von zerstörerischen Eingriffen in diese Umwelt häufig überhaupt erst ermöglicht wurde (Bond 2013; Masco 2010). Dadurch bekommt Vicos *verum factum* – dass sich nur das wirklich erkennen lässt, was selbst hergestellt wurde – eine neue epistemologische Virulenz. Allerdings wird das Herstellen immer häufiger zum Zerstören, die viel beschworene „Performativität“ der Technowissenschaften erweist sich wieder einmal zu allererst als deformativ (Folkers 2020).

Was dem Beitrag von Warde, Robin und Sörlin gegenüber dem von Bonneuil und Fressoz fehlt ist eine scharfe kritische Perspektive auf die politische Epistemologie „der Umwelt“ und des Anthropozäns. Denn der geokratische Macht-Wissenskomplex des Anthropozäns ist aus einer Reihe von Gründen problematisch. Erstens, soll eben doch wieder eine Expertokratie aus Erdsystem- bzw. Klimawissenschaften die Kommandobrücke des Raumschiffs Erde okkupieren. Das wird weder dadurch besser, dass es zumindest zum Teil auch durch soziale Bewegungen eingefordert wird, noch dadurch, dass bisweilen „die Wissenschaft“ weniger mit einzelnen Expert*innen, sondern eher mit einer komplexen Infrastruktur identifiziert wird, die eine Vielzahl reflexiver Öffentlichkeit in-formieren soll (Lenton & Latour 2018). Es ist allzu naiv anzunehmen, dass die Wissenschaftsinfrastrukturen des Anthropozäns nicht auch tiefsitzende Machtstrukturen reproduzieren.^3^ Zweitens marginalisiert die Vorherrschaft eines bestimmten Kanons anerkannter Wissensformen alternative Arten des Wissens um ökologische Zusammenhänge (Moreno et al. 2015). Das gilt für indigenes Wissen (Davis & Todd 2017; Spencer et al., 2019) ebenso wie für „grammars of environmental reflexivity“ (Bonneuil & Fressoz 2016: 170–197), im Marxismus (Foster & Bellamy 2000), Ökofeminismus (Merchant 1987) und der radikalen Umweltbewegung, die schon lange vor der gegenwärtigen Erdsystemwissenschaft ein ökologisches Krisenbewusstsein entwickelt haben. Drittens ist die zeitliche Dramaturgie der geokratischen Erzählung dazu angelegt, sämtliche Fragen historischer Verantwortung für ökologische Probleme der Gegenwart wie den „unequal ecological exchange“ (Hornborg 2009) zwischen Nord und Süd und die verhängnisvollen imperialen Ursprünge des Anthropozäns (Folkers 2020) auszublenden.

Dagegen eröffnen sich aus den oben diskutierten alternativen erdhistorischen Konzepten in den Sozial- und Kulturwissenschaften deutlich nuanciertere Verantwortungsverständnisse und politische Handlungsoptionen. Verantwortlich für das Kapitalozän oder das Plantationozän ist eben nicht „die Menschheit“, sondern historisch spezifische Herrschafts- und Ausbeutungsverhältnisse wie Kolonialismus, Rassismus und Kapitalismus. Der gewöhnliche emanzipatorische Reflex ist bekanntlich auf solch eine Forderung zu reagieren, indem man die Abschaffung dieser Verhältnisse fordert. Gleichwohl ist in diesem Fall klar, dass sich diese Herrschaftsstrukturen so tief in den Planeten eingeschrieben haben, dass sie auch dann noch von erdgeschichtlicher Bedeutung sein werden, wenn sie sozialgeschichtlich bereits beendet sein sollten. Es geht also nicht nur darum, die ökologische Katastrophe in der Zukunft abzuwenden, sondern mit den bereits eingetretenen Zerstörungen und deren langwierigen Folgen umzugehen. So sieht Anna Tsing (2015) im Anthropozän die Ruinen des Kapitalismus und sucht gemeinsam mit den Autor*innen eines sehr lesenswerten Sammelbands nach den „arts of living on a damaged planet“ (Tsing et al. 2017). Der Kapitalismus hinterlässt verwüstete Landschaften, die für deren Bewohner*innen menschlicher und nicht-menschlicher Art große Herausforderungen mit sich bringen. So gilt es mit vielen ökologischen Gespenstern zu leben, also etwa bereits ausgestorbenen Tier- und Pflanzenarten, die sich gerade durch ihre Abwesenheit in den „haunted landscapes of the Anthropocene“ (Tsing et al. 2017: G1–G14) bemerkbar machen.

Ähnlich argumentiert auch Kathrin Yusoff (2018: 106) sowohl gegen eine zukunftsfixierte Anthropozänerzählung wie auch gegen die Geologie von Charles Lyell, für den die abgelagerte geologische Gegenwart lediglich der Schlüssel zur Vergangenheit war. Vielmehr gelte es heute anzuerkennen, „that the key to the future lies in the present and that the present is not just future oriented but that the future is already inscripted in the present“. Der Zusammenhang von Rassismus und ökologischen Problemen setzt sich auch nach dem Ende von Sklaverei und Kolonialismus fort – etwa im „environmental racism“ (Bullard 1993) und der grundlegenden historischen Ungerechtigkeit, dass die Länder des Globalen Südens zwar weitaus weniger zum Klimawandel beigetragen haben, aber viel stärker von dessen Auswirkungen betroffen sind. Aus solchen Reflexionen über das komplexe Verhältnis von Vergangenheit, Gegenwart und Zukunft in der Erdgeschichte lassen sich politische Forderungen ableiten, die über generische Konzeptionen einer Anthropozänethik hinausgehen (Karera 2019). Statt bloß für eine universelle „responsibility“ zu plädieren, müssten konkrete Formen sozio-ökologischer „redistribution“ und „reappropriation“ eingefordert werden (Folkers 2020). Eine solche Umverteilung müsste sowohl Reparationen für vergangenes Unrecht (Patel & Moore 2017: 208–210) als auch „reimbursements“ für die in Zukunft anfallenden Reparaturkosten auf dem beschädigten Planeten beinhalten. All diese Fragen sind dabei immer sowohl mit enormen ethischen als auch epistemologischen Herausforderungen verbunden, weil jeweils überhaupt erst bestimmt werden muss, was wie umzuverteilen ist und was wie entschädigt werden kann. Wiederum zeigt sich, dass das Anthropozän weniger eine Epoche als eine Zeit des Übergangs ist (Haraway et al. 2016: 541), deren Sinn sich erst vollends im Ausgang dieser Krisenperiode erweisen wird. Was das Erdzeitalter gewesen sein wird, steht also noch lange nicht fest. Das anhängige Verfahren läuft noch und eine Vielzahl menschlicher und nicht-menschlicher Zeug*innen wartet noch darauf, gehört zu werden.

## Footnotes

1 Insofern ist es nur konsequent wenn der Geologe Peter Haff (2014) gar nicht mehr „die Menschheit“, sondern die „Technosphäre“ als entscheidendes Moment der Transformation des Planten ausmacht. Die Technosphäre wird dabei als eine von menschlicher Intention relativ autonome Totalität technischer Artefakte und Infrastrukturen verstanden, die so wie die Bio‑, Hydro‑, Atmo- oder Lithosphäre als eine genuine Ökosphäre gelten muss. Der Hauptunterschied zu den anderen Sphären ist, dass die Technosphäre auf nicht-erneuerbare Ressourcen (insbesondere Energie) zurückgreifen muss und ihre Abfallprodukte nicht von anderen Sphären recycelt werden können. Siehe auch Edwards & Hecht (2016).

2 Zur Ethik der Verantwortung auf der und für die Erde innerhalb der feministischen Theorie, siehe: (Folkers & Marquardt 2017; Hoppe 2019).

3 Dieser Aspekt kommt bei Edwards (2015) deutlich zu kurz. So sieht er auch in der Logistik von IKEA oder den Informationsflüssen im Finanzwesen Momente der Wissensinfrastruktur des Anthropozäns, ohne zu reflektieren, dass in diesen Infrastrukturen ökonomistische und neoliberale Wissensmythen verbaut sind (Folkers 2019).

## References

[CR1] Biermann F (2012). Navigating the Anthropocene: improving earth system governance. Science.

[CR2] Bond D (2013). Governing disaster: the political life of the environment during the BP oil spill. Cultural Anthropology.

[CR3] Bonneuil C, Fressoz J-B (2016). The shock of the Anthropocene: the earth, history and us.

[CR4] Bullard RD (1993). Confronting environmental racism: voices from the grassroots.

[CR5] Chakrabarty D (2009). The climate of history: four theses. Critical Inquiry.

[CR6] Clark N (2012). Rock, life, fire: speculative geophysics and the Anthropocene. Oxford Literary Review.

[CR7] Clark N, Yusoff K (2017). Geosocial formations and the Anthropocene. Theory, Culture & Society.

[CR8] Crutzen PJ (2002). Geology of mankind. Nature.

[CR9] Davis H, Todd Z (2017). On the importance of a date, or decolonizing the Anthropocene. ACME: an international E-journal for critical. Geographies.

[CR10] Davis H, Turpin E (2015). Art in the Anthropocene: encounters among aesthetics, politics, environments and epistemologies.

[CR11] Davis J, Moulton A, Van Sant L, Williams B (2019). Anthropocene, Capitalocene,… Plantationocene?: a manifesto for ecological justice in an age of global crises. Geography Compass.

[CR12] Edwards PN (2010). A vast machine: computer models, climate data, and the politics of global warming.

[CR13] Edwards PN, Renn J, Scherer B (2015). Wissenschaftsinfrastrukturen für das Anthropozän. Das Anthropozän: Zum Stand der Dinge.

[CR14] Edwards, Paul N. und Gabrielle Hecht 2016. Taking on the Technosphere. A Kitchen Debate. Technosphere Magazine. https://technosphere-magazine.hkw.de/p/Taking-on-the-Technosphere-A-Kitchen-Debate-4TGo3PL5LWM7JydaVdQPHC. Letzter Zugriff: 12.7.2018.

[CR17] Folkers A, Marquardt N, Bath C, al (2017). Die Kosmopolitik des Ereignisses. Gaia, das Anthropozän und die Welt ohne uns. Verantwortung und Un/Verfügbarkeit. Impulse und Zugänge eines (neo)materialistischen Feminismus.

[CR18] Folkers A, Marquardt N (2018). Die Verschränkung von Umwelt und Wohnwelt. Grüne smart homes aus der Perspektive der pluralen Sphärologie. Geographica Helvetica.

[CR79] Folkers, Andreas und Sven Opitz 2019. Symbiose als Begriff und Gegenstand der Soziologie. In: Burzan, Nicole (Hg.). Komplexe Dynamiken globaler und lokaler Entwicklungen. Verhandlungen des 39. Kongresses der Deutschen Gesellschaft für Soziologie in Göttingen 2018.

[CR80] Folkers, Andreas 2019. Smart grids and smart markets. The promises and politics of intelligent infrastructures. In: Martin Kornberger, Julia Elyachar, Peter Miller, Geoffrey Bowker, Andrea Mennicken, Neil Pollock (Hg.) Thinking Infrastructure. Research in the Sociology of Organizations. Bingley: emeraldinsight.

[CR15] Folkers, Andreas 2020. Air-appropriation: The imperial origins and legacies of the Anthropocene. European Journal of Social Theory: Onlinefirst. 10.1177/1368431020903169.

[CR20] Foster, Bellamy J (2000). Marx’s ecology: materialism and nature.

[CR21] Foucault M (1974). Die Ordnung der Dinge.

[CR22] Gabrys J (2016). Program earth: environmental sensing technology and the making of a computational planet.

[CR23] Gilbert SF, Lowenhaupt Tsing A, al (2017). Holobiont by birth. Multilineage individuals as the concretion of cooperative processes. Arts of living on A damaged planet: ghosts and monsters of the Anthropocene.

[CR24] Haff PK (2014). Technology as a geological phenomenon: implications for human well-being.

[CR25] Hamilton C (2015). Getting the Anthropocene so wrong. The Anthropocene Review.

[CR26] Haraway D (2016). Staying with the trouble: making kin in the Chthulucene.

[CR27] Haraway, Noboru Ishikawa DSFG, Olwig K, Tsing AL, Bubandt N (2016). Anthropologists are talking—about the Anthropocene. Ethnos.

[CR28] Hecht G (2018). Interscalar Vehicles for an African Anthropocene: On Waste, Temporality, and Violence. Cultural Anthropology.

[CR29] Henkel A, Laux H (2018). Die Erde, der Mensch und das Soziale. Zur Transformation gesellschaftlicher Naturverhältnisse im Anthropozän.

[CR30] Hoppe K (2019). Responding as composing: towards a post-anthropocentric, feminist ethics for the Anthropocene. Distinktion: Journal of Social Theory.

[CR31] Horn, Eva. 2016. Klimatologie um 1800. Zur Genealogie des Anthropozäns. Zeitschrift für Kulturwissenschaften. Heft 1/2016: 87–102.

[CR32] Hornborg A (2009). Zero-sum world: challenges in conceptualizing environmental load displacement and ecologically unequal exchange in the world-system. International Journal of Comparative Sociology.

[CR33] Jasanoff S, Jasanoff S, Martello ML (2004). Heaven and earth. The politics of environmental images. Earthly politics: local and global in environmental governance.

[CR34] Karera A (2019). Blackness and the pitfalls of Anthropocene ethics. Critical Philosophy of Race.

[CR35] Kersten J (2014). Das Anthropozän-Konzept. RW Rechtswissenschaft.

[CR36] Koselleck R (2000). Zeitschichten: Studien zur Historik.

[CR37] Latour B (2017). Facing Gaia. Eight lectures on the new climatic regime.

[CR39] Lenton TM, Latour B (2018). Gaia 2.0. Science.

[CR38] Lenton TM, Held H, Kriegler E, Hall JW, Lucht W, Rahmstorf S, Schellnhuber HJ (2008). Tipping elements in the earth’s climate system. Proceedings of the National Academy of Sciences.

[CR40] Lewis SL, Maslin MA (2015). Defining the Anthropocene. Nature.

[CR41] Lovelock JE, Margulis L (1974). Atmospheric homeostasis by and for the biosphere: the gaia hypothesis. Tellus.

[CR42] Luisetti F (2018). Geopower: on the states of nature of late capitalism. European journal of social theory.

[CR43] Margulis L (1999). Symbiotic planet: a new look at evolution.

[CR44] Masco J (2010). Bad weather: on planetary crisis. Social Studies of Science.

[CR45] McFall-Ngai M, Lowenhaupt Tsing A, al (2017). Noticing microbial worlds. The postmodern synthesis in biology. Arts of living on a damaged planet: ghosts and monsters of the Anthropocene.

[CR46] McKittrick K (2013). Plantation futures. Small Axe: A Caribbean Journal of Criticism.

[CR47] Meillassoux Q (2008). Nach der Endlichkeit: Versuch über die Notwendigkeit der Kontingenz.

[CR48] Merchant C (1987). Der Tod der Natur. Ökologie, Frauen und die neuzeitliche Naturwissenschaft.

[CR49] Moore JW (2015). Capitalism in the web of life: ecology and the accumulation of capital.

[CR50] Moreno C, Fuhr L, Speich Chassé D (2015). Carbon metrics: glocal abstractions and ecological epistemicide.

[CR51] Parikka J (2014). The Anthrobscene.

[CR52] Patel R, Moore JW (2017). A history of the world in seven cheap things. A guide to capitalism, nature, and the future of the planet.

[CR53] Pottage A, Desautels-Stein J, Tomlins C (2017). Our geological contemporary. Searching for contemporary legal thought.

[CR54] Pottage A (2019). Holocene Jurisprudence. Journal of Human Rights and the Environment.

[CR56] Rothe D, Harker M, Eroukhmanoff C (2017). Global security in a posthuman age? IR and the Anthropocene challenge. Reflections on the posthuman in international relations.

[CR57] Schellnhuber H-J (1999). ‘Earth system’ analysis and the second Copernican revolution. Nature.

[CR58] Schroer M (2015). Erde, Klima, Territorium. Konturen einer Geosoziologie. Merkur. Zeitschrift für europäisches Denken.

[CR60] Serres M (1994). Hermes: Die Nordwest-Passage.

[CR61] Sloterdijk P, Renn J, Scherer B (2015). Das Anthropozän – ein Prozess-Zustand am Rande der Erd-Geschichte?. Das Anthropozän: Zum Stand der Dinge.

[CR62] Spencer M, Dányi E, Hayashi Y (2019). Asymmetries and climate futures: working with waters in an indigenous Australian settlement. Science, Technology, & Human Values.

[CR63] Sprenger F (2019). Epistemologien des Umgebens. Zur Geschichte, Ökologie und Biopolitik künstlicher ‚environments‘.

[CR65] Steffen W, Broadgate W, Deutsch L, Gaffney O, Ludwig C (2015). The trajectory of the Anthropocene: the great acceleration. The Anthropocene Review.

[CR64] Steffen W, Persson Å, Deutsch L, Zalasiewicz J, Williams M, Richardson K, Crumley C, Crutzen P, Folke C, Gordon L, Molina M, Ramanathan V, Rockström J, Scheffer M, Schellnhuber HJ, Svedin U (2011). The Anthropocene: from global change to planetary stewardship. Ambio.

[CR67] Tsing, Lowenhaupt A (2015). The mushroom at the end of the world: on the possibility of life in capitalist ruins.

[CR68] Tsing, Lowenhaupt A, Swanson HA, Gan E, Bubandt N (2017). Arts of living on a damaged planet: ghosts and monsters of the Anthropocene.

[CR69] Wakefield S (2014). The crisis is the age. Progress in Human Geography.

[CR70] Wark MK (2015). Molecular red: theory for the Anthropocene.

[CR71] Westermann A, Edelstein D, Stefanos Geroulanos, Wheatley N (2020). A technofossil of the Anthropocene. Sliding up and down temporal scales with plastic. Power and time. Temporalities in conflict and the making of history.

[CR72] Wynter S (2003). Unsettling the Coloniality of Being/Power/Truth/Freedom: Towards the Human, After Man, its Overrepresentation—An Argument. The New Centennial Review.

[CR73] Young OR, Steffen W, Chapin SF, Folke C, Kofinas GP (2009). The earth system: sustaining planetary life-support systems. Principles of ecosystem stewardship. Resilience-based natural ressource management in a changing world.

[CR74] Yusoff K (2016). Anthropogenesis: origins and endings in the Anthropocene. Theory, Culture & Society.

[CR75] Yusoff K (2018). A billion black Anthropocenes or none.

[CR76] Zalasiewicz J, Renn J, Scherer B (2015). Die Einstiegsfrage: Wann hat das Anthropozän begonnen?. Das Anthropozän: Zum Stand der Dinge.

[CR78] Zalasiewicz J (2017). Scale and diversity of the physical technosphere: a geological perspective. The Anthropocene Review.

[CR77] Zalasiewicz J, Williams M, Waters CN, Barnosky AD, Haff P (2014). The technofossil record of humans. The Anthropocene Review.

[CR55] Renn J, Scherer BM (2015). Das Anthropozän: Zum Stand der Dinge.

